# Rates of parent‐reported allergic conditions in children at‐risk of celiac disease

**DOI:** 10.1002/jpr3.70079

**Published:** 2025-08-19

**Authors:** Timothy Sun, Victoria Kenyon, Francesco Valitutti, Alessio Fasano, Victoria Martin, Maureen M. Leonard

**Affiliations:** ^1^ Pediatric Allergy Immunology, Mass General Hospital for Children Harvard Medical School Boston Massachusetts USA; ^2^ Center for Celiac Research and Treatment, Mass General Hospital for Children Harvard Medical School Boston Massachusetts USA; ^3^ Department of Medicine and Surgery, Pediatric Unit University of Perugia Perugia Italy; ^4^ Pediatric Gastroenterology, Mass General Hospital for Children Harvard Medical School Boston Massachusetts USA

**Keywords:** allergy, autoimmune, cow's milk protein allergy, food allergy, immune

## Abstract

Allergies and other chronic immune mediated conditions are becoming increasingly common. Here we utilized a prospective birth cohort called the Celiac Disease Genomic Environmental Microbiome and Metabolomic (CDGEMM) study to examine the frequency of parent reported allergic conditions and their association with celiac disease (CeD). We examined 271 children at‐risk of CeD from the United States and found a high frequency of allergic conditions. In our overall cohort, 19.8% reported food protein‐induced allergic proctocolitis (FPIAP), 12.5% reported IgE‐mediated food allergy, and 14.7% reported atopic dermatitis. Among the 23 children with CeD, 21.74% had FPIAP, 8.7% had an IgE‐mediated food allergy, and 21.74% had atopic dermatitis. No significant association between allergic conditions and CeD was found (*p* > 0.35 for all). These results highlight the widespread occurrence of parent‐reported allergic conditions in children but do not suggest an association between allergic conditions and CeD development.

## Abstract (highlights)

**What is Known:**

- The incidence and prevalence of allergic and other immune mediated diseases, such as celiac disease, are rising.
- Development of allergic conditions play a role in early immune system dysregulation.
- Allergic conditions can increase the likelihood of developing other allergic conditions over time.

**What is New:**

- We identified a high prevalence of parent‐reported allergic conditions in infants at‐risk for celiac disease.
- The frequency of food protein‐induced allergic proctocolitis was 19.8%, which is similar to previously described prevalence in a healthy cohort.
- We did not find an association between developing celiac disease and having an allergic condition in early childhood.

## 1 INTRODUCTION

Allergic conditions, such as seasonal allergies, immunoglobulin E (IgE) mediated food allergies, and atopic dermatitis (AD), are becoming increasingly more common. In the United States (US), recent studies in children have found that 18.9% had a seasonal allergy, 10.8% had eczema, and 5.8% had a food allergy, totaling 27.2% with at least one of these conditions.^1^ These allergic conditions often emerge in infancy, with AD frequently serving as the first manifestation of what is known as the atopic march, a term used to describe the progression of how early allergic conditions can increase the likelihood of developing other allergic conditions over time.^2^

In addition to skin‐related allergic conditions, food allergies play a significant role in early immune system dysregulation. One of the earliest manifestations of food allergy in infancy is food protein‐induced allergic proctocolitis (FPIAP), also referred to as cow's milk protein allergy or intolerance, non‐IgE‐mediated cow's milk allergy, or milk/soy protein intolerance. Analogous to AD in the skin, FPIAP is thought to be caused by a disruption in the intestinal barrier of infants exacerbated by exposure to common allergens (most commonly milk and soy).^3^

While allergic conditions have been shown to be increasing, studies also support a rise in immune mediated conditions such as celiac disease (CeD),^4^ type 1 diabetes,^5^ and inflammatory bowel disease.^6^ There is limited data examining the association between allergic and chronic inflammatory conditions; however, it has been suggested that allergic conditions may serve as a potential risk factor for the development of CeD.^7^

Nearly 20% of infants may be diagnosed with FPIAP in the United States,^3^ and we sought to validate those reported rates in this independent birth cohort of children at risk for CeD across the United States. We also aimed to examine whether a diagnosis of FPIAP in early infancy was associated with an increased risk of CeD by age 5, given the distinct but analogous hypothesized pathogenesis of these two non‐IgE‐mediated entities. To determine these prevalence rates, we utilized an ongoing longitudinal birth cohort study called the Celiac Disease Genomic Environmental Microbiome and Metabolomic (CDGEMM) study.

## 2 METHODS

The CDGEMM study enrolls infants 0–6 months of age at‐risk for CeD due to having a first‐degree family member with CeD.^8^ Subjects are tested for CeD every 6 months for the first 3 years after birth and then tested yearly using serology for Immunoglobulin A (IgA) tissue transglutaminase (tTG) and Immunoglobulin G (IgG) deamidated gliadin peptide (DGP). Subjects are diagnosed with CeD according to biopsy criteria,^9^ nonbiopsy criteria,^10^ or if IgA tTG is elevated on two occasions. Parent‐reported surveys with questions related to diet, medical history, symptoms, and lifestyle factors are collected monthly in the first year of life and then every 3–6 months until age 5. As it relates to allergic conditions, parents are asked to report if their child was diagnosed with eczema, cow milk protein intolerance, soy protein intolerance, and/or an IgE‐mediated food allergy. In addition to the questionaries, children in the study are tested every 6 months until age 3 and then every year until age 5 for CeD antibodies (tissue transglutaminase IgA, deaminated gliadin IgG, and/or endomysial antibody IgA) to determine CeD autoimmune status.

Data was captured in REDCap (Research Electronic Data Capture) at MassGeneral Brigham. Survey data was analyzed using R v4.3.0 (tidyverse 2.0.0 and dplyr 1.2.1). The data analyzed was collected between August 2014 and June 2024. The average age (in months) of children at the time of these analyses was 72 months (range: 3–123), and 186/273 (68%) were >60 months. Study physicians reviewed parent‐reported information concerning reactions to consumption of potential allergens and identified likely IgE‐mediated food allergies as having a combination of positive skin testing and consistent symptoms, or multisystem presentation (e.g., anaphylaxis, or eg hives and vomiting). Further, parents who reported their child had eczema and/or milk or soy protein intolerance were included as such. After categorization, we summarized the overall proportions of these IgE‐ and non‐IgE‐mediated allergic conditions and compared (using fisher's exact testing) rates in children with and without CeD.

### 2.1 Ethics statement

The MassGeneral Brigham Institutional Review Board (IRB) approved the study (Protocol ID #2013P001965). Consent was obtained by parent/guardian before participation. This study is registered on clinicaltrial.gov (Clinical Trial Identifier: NCT02061306).

## 3 RESULTS

**Figure 1:**
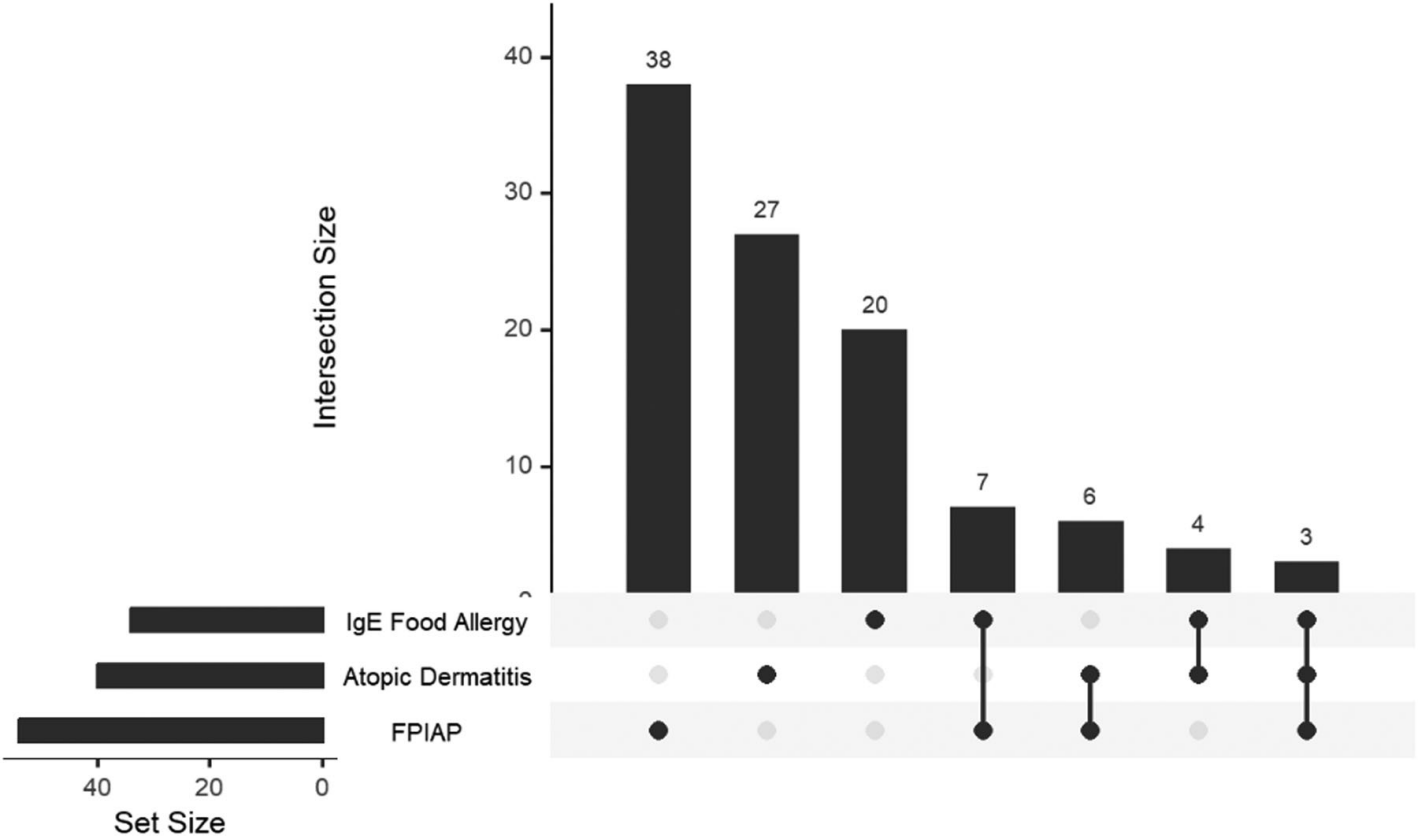
Intersection of common allergic conditions. Set size (horizontal bars) indicates total number of infants with each individual condition. Intersection size (vertical bars) indicates number of infants with each combination of conditions. FPIAP, food protein‐induced allergic proctocolitis.

We analyzed a total of 271 children from the US who were at‐risk for CeD (49.5% female) (Table 1). In the non‐CeD group (*n* = 248), we found that 19.8% reported FPIAP, 12.5% reported a possible IgE‐mediated food allergy, and 14.7% reported eczema or AD. Additionally, 168 (61.5%) were negative for all three conditions. In the CeD group (*n* = 23), we found that 2 (8.7%) reported IgE‐mediated food allergy, 5 (21.74%) reported FPIAP, and 5 (21.74%) reported AD. The CeD group was further categorized as biopsy proven (*n* = 17) and serology (*n* = 6). We did not find a significant association between IgE‐mediated food allergy, FPIAP, or AD and a diagnosis of CeD (*p* = 0.750, 0.787, and 0.352 respectively). The intersection of the IgE‐mediated food allergy, FPIAP, and AD is shown in Figure 1.

**Table 1:** Demographics of infants analyzed from this cohort stratified by CeD status.

	Non‐CeD Cohort: *n* (%)	CeD Cohort: *n* (%)
	248	23
Gender		
Female	118 (47.5%)	17 (73.9%)
Race		
White	229 (92.3%)	22 (95.7%)
Black	0 (0.0%)	0 (0.0%)
Asian	0 (0.0%)	1 (4.3%)
Not reported	3 (1.2%)	0 (0.0%)
More than 1 race	16 (6.5%)	0 (0.0%)
Ethnicity		
Hispanic or Latino	9 (3.6%)	0 (0.0%)
Delivery mode		
C‐section	80 (32.3%)	1 (4.3%)
Infant diet (0–12 months)		
Formula	40 (16.1%)	5 (21.7%)
Breastmilk	139 (56.0%)	10 (43.5%)
Mixed	62 (25.0%)	8 (34.8%)
Unknown	7 (2.9%)	0 (0.0%)
Allergic condition		
FPIAP	49 (19.80%)	5 (21.74%)
IgE‐FA	35 (14.10%)	2 (8.7%)
Atopic dermatitis	32 (12.90)	5 (21.74%)

Abbreviations: CeD, celiac disease; FPIAP, food protein‐induced allergic proctocolitis; IgE‐FA, immunoglobulin E mediated food allergies.

## 4 DISCUSSION

We identified a high prevalence of parent‐reported allergic conditions in our prospective observational cohort of infants at‐risk for CeD. Our results are comparable to the those reported by the GMAP cohort who found a 17% incidence of FPIAP, 6.2% incidence of confirmed IgE‐mediated food allergy, and 43% incidence of eczema.^3^ Specifically, 13.6% of children in the cohort, including both CeD and non‐CeD groups, reported an IgE‐mediated food allergy. This rate is twice that reported by the Centers for Disease Control^1^ but it aligns with other research that reported high rates of food allergies in the US based on parent‐reported data, rather than physician diagnoses.^11^ Additionally, the rate of FPIAP in this cohort was 19.8%, which is similar to the 17% prevalence previously reported from the GMAP cohort (a prospective observational healthy infant cohort study designed to identify FPIAP in suburban Massachusetts).^12^ Nearly 15% of parents reported that their children had eczema or AD, which is consistent with prior reports of high rates of CeD and AD.^13^ Despite these findings, we did not find an association between developing CeD and having an allergic condition in early childhood.

Limitations of our study include the method by which the survey data was collected. Specifically, the data relied heavily on parent‐reported information, and in some instances, responses were provided in free‐text fields. As a result, the reported prevalence rates of the above allergic conditions may be higher than what would be observed if a formal clinical diagnosis with supportive testing or challenge were required. Survey data collected between August 2014 and June 2024 was analyzed, and given the study is ongoing and subjects are actively in the study, subjects had a varying number of surveys completed. Additionally, this study is ongoing and thus subjects could develop CeD later. The average age at time of seroconversion is 33‐months, whereas the average age of the cohort is 48‐months. While we project that most subjects will seroconvert by age 36 months, delayed seroconversion may occurIn addition, while our cohorts are large, our sample size of subjects with CeD and allergy is low and therefore larger sample sizes we may be necessary to examine associations.

## 5 CONCLUSION

Rising rates of FPIAP, AD, IgE‐mediated food allergies, and autoimmune diseases, such as CeD, in our nation's children are important areas for prospective research. In this study, there was no association between CeD and other parent‐reported allergic conditions. However, FPIAP, IgE‐FA, and CeD are all diseases which can begin in early childhood and where genetic risk, microbiome composition, and permeability of the infant gastrointestinal tract likely drive antigen/allergen (mis)trafficking and result in untoward reaction to a food. Some of these can be transient and outgrown, while others are lifelong. More prospective mechanistic research into the details of these analogous yet distinct pediatric diseases, particularly understanding what leads to tolerance acquisition versus life‐long disease, has great potential to identify opportunities for treatment, cure, and even prevention.

## CONFLICT OF INTEREST STATEMENT

Victoria Kenyon reported acting as a consultant for Takeda. Maureen M. Leonard reported receiving research support from Takeda Pharmaceuticals and Moderna Inc., and acting as a consultant for Takeda, Anokion, and EllaOla. Victoria M. Martin serves as a consultant and on the Scientific Advisory Board of Milk Care Co. AF serves as Chief Science and Medical Officer at Mead Johnson Nutrition. Other authors have declared that no conflicts of interest exist. None of the above financial disclosures are related to this study.
